# An integrated strategy of multiplex qPCR and *BclA* gene length polymorphism analysis for differentiating *Bacillus anthracis* wild-type and vaccine strains

**DOI:** 10.1128/spectrum.00192-26

**Published:** 2026-09-01

**Authors:** Jiaqi Yang, Yan Liang, Bing Liang, Yanfang Li, Lingwei Zhu, Yonggang Qu

**Affiliations:** 1 College of Animal Science and Technology, Shihezi University 70586 , Shihezi, Xinjiang, China; 2 Changchun Veterinary Research Institute, Chinese Academy of Agricultural Sciences 12661 , Changchun, Jilin, China; Ann & Robert H. Lurie Children's Hospital of Chicago, Chicago, Illinois, USA; Beijing Institute of Biotechnology, Beijing, China

**Keywords:** *Bacillus anthracis*, strain differentiation, multiplex real-time PCR, *BclA *gene, length polymorphism analysis

## Abstract

A combined molecular strategy was developed to accurately differentiate wild-type and vaccine strains of *Bacillus anthracis*. This approach integrates a multiplex TaqMan qPCR assay targeting the *BclA*, *sap*, and *cap* genes with conventional PCR analysis of *BclA* fragment length polymorphism. The qPCR assay demonstrated high specificity, with no cross-reactivity against other common pathogens, and exhibited a consistent limit of detection of 2.11 × 10^1^ copies/μL (Cq < 35) for all three targets. Excellent repeatability was confirmed, with intra- and inter-assay coefficients of variation below 1.96% and 3.06%, respectively. Validation using 19 wild-type strains revealed two distinct qPCR profiles, and all wild-type strains were conclusively differentiated from the capsule-producing Chinese vaccine strain II and the acapsular Sterne strain by their characteristic *BclA* amplicon sizes. Through simulation PCR validation of common global canSNP genotyping (groups A, B, and C), it is possible to distinguish between wild-type and vaccine strains in China. This integrated “multiplex qPCR–BclA length analysis” protocol provides a reliable, sensitive, and specific tool for the precise detection and differentiation of *B. anthracis* strains in China.

**IMPORTANCE:**

Anthrax, which is caused by the bacterium *B. anthracis*, remains a serious zoonotic disease. Its effective surveillance relies on precise strain identification. Currently, it is challenging to rapidly and accurately distinguish wild-type strains from vaccine strains, which hinders outbreak investigation and response. This study develops a novel molecular strategy combining multiple genetic targets to solve this critical diagnostic problem. The new method provides a reliable tool for strain differentiation, directly supporting accurate source tracing and informed decision-making during anthrax outbreaks. By improving diagnostic precision, this work contributes to strengthening public and veterinary health defenses against this persistent threat.

## INTRODUCTION

Anthrax, which is caused by *B. anthracis*, is a severe acute zoonotic disease affecting both humans and livestock. As a spore-forming bacterium, *B. anthracis* can persist in the environment for decades, posing a persistent threat (1). Ruminants, such as cattle, sheep, and goats, are the primary susceptible hosts typically infected via ingestion, inhalation, or cutaneous exposure. Human cases usually result from contact with infected animals, contaminated products, or spores (1). Anthrax is present in most parts of the world, but the frequency of outbreaks varies, with most cases concentrated in rural rainfed systems, where it poses a major threat to both environmental safety and public health (2).

The virulence of *B. anthracis* primarily depends on two plasmids: pXO1 encoding the tripartite toxin complex and pXO2 that carries the cap gene cluster (encoding the poly-D-glutamate capsule). The chromosome harbors key genes, such as *sap*, encoding S-layer proteins, and *BclA*, responsible for the exosporium’s hair-like nap. Notably, genetic variations, particularly in the *BclA* gene, have been reported among different strains (3–5). The *cap*, *sap*, and *BclA* genes are often targeted for the molecular detection of this pathogen.

Current laboratory diagnostics for anthrax include bacterial culture, immunological assays, and various nucleic acid amplification techniques (e.g., PCR, qPCR, and isothermal amplification) (6–11). While effective for species confirmation, most existing methods lack the discriminatory power to differentiate between wild-type and attenuated vaccine strains. This limitation is particularly critical in regions like China, where live-attenuated vaccines are extensively used for animal immunization. The widely used Chinese vaccine strain II (CVCC40202, pXO1**^+^**, pXO2**^+^**, A.Br.008/009) introduced from the former Soviet Union and the acapsular Sterne strain 34F2 (CVCC40205, pXO1^+^, pXO2^−^, A.Br.001/002) can persist as spores in the environment following vaccination or potential leakage. Consequently, it becomes impossible to determine whether detected spores originate from a natural outbreak or historical vaccine use, severely hampering outbreak investigation and source attribution.

To address this diagnostic gap, we developed an integrated molecular strategy. A multiplex TaqMan qPCR assay simultaneously targeting the *sap*, *cap*, and *BclA* genes was established, coupled with *BclA* gene length polymorphism analysis. This combined approach was rigorously validated for specificity, sensitivity, and reproducibility to provide a reliable tool for precise strain differentiation, thereby supporting accurate epidemiological tracing and informed anthrax control.

## RESULTS

### Preparation of plasmid standards

The recombinant plasmid standards (pMD19-BclA, pMD19-cap, and pMD19-sap) were quantified, yielding concentrations of 161.1, 219.7, and 311.0 ng/μL corresponding to 4.32 × 10^10^, 5.67 × 10^10^, and 8.52 × 10^10^ copies/μL, respectively. The integrity and specificity of each plasmid were confirmed by PCR using the same gene-specific primers employed in the detection assays, with agarose gel electrophoresis showing single amplicons of the expected size (Fig. 1, lanes 2, 5, and 8). The identity of the cloned inserts was definitively verified by Sanger sequencing (sequence data available from the authors upon reasonable request). These validated plasmids were subsequently used as standards for all optimization and validation experiments of the multiplex qPCR assay.

**Fig 1:**
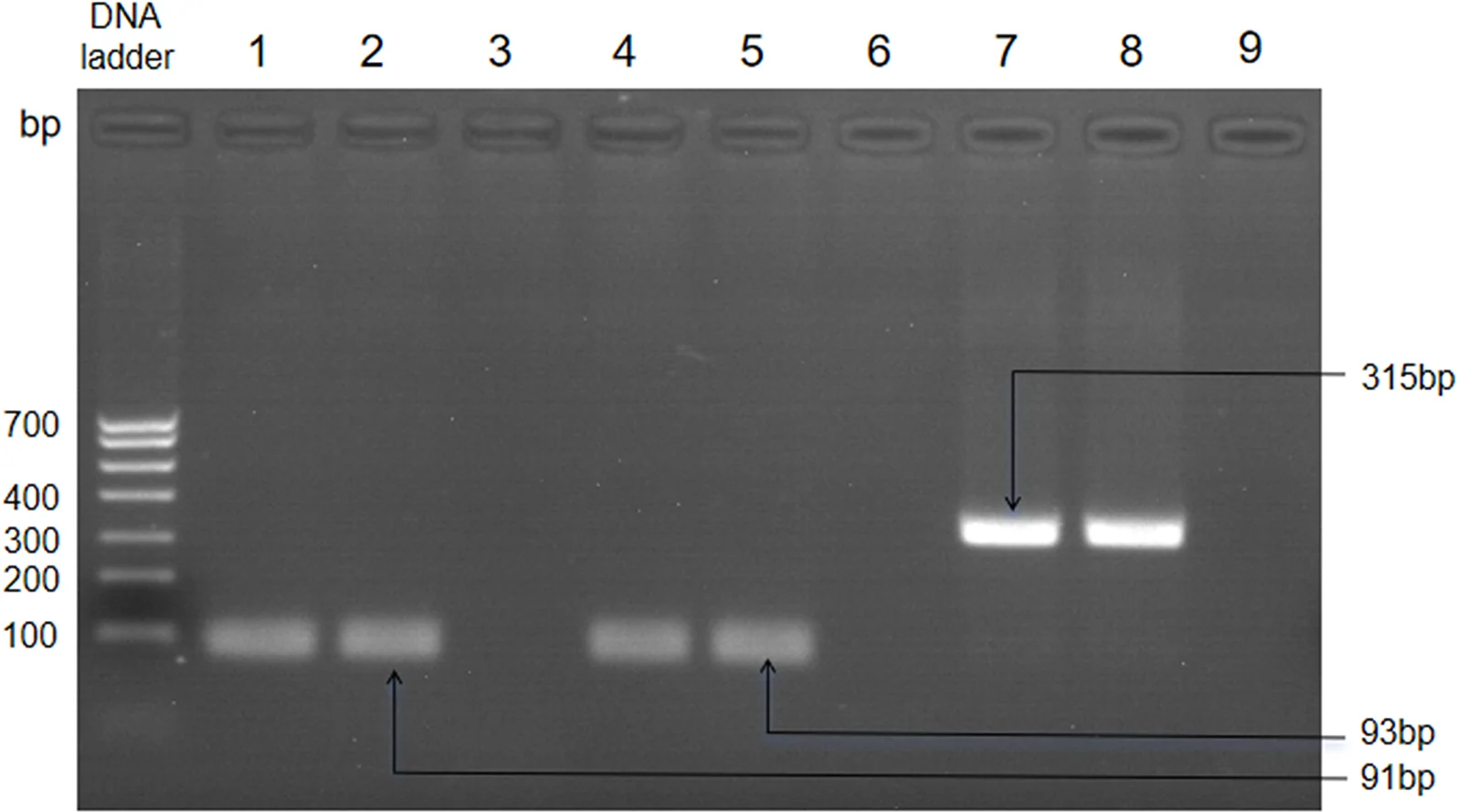
Verification of recombinant plasmid standards by PCR. The integrity and specificity of each plasmid construct were confirmed by PCR using gene-specific primers. Expected amplicons were observed for all three targets: Lane 2, pMD19-sap; Lane 5, pMD19-cap; Lane 8, pMD19-BclA. Lanes 1, 4, and 7 contain amplicons from Chinese vaccine strain II DNA, serving as positive controls for the *sap*, *cap*, and *BclA* genes, respectively. Lanes 3, 6, and 9 represent the negative control for the *sap*, *cap*, and *BclA* genes (nuclease-free water), respectively.

### Optimization of the multiplex qPCR assay

Following systematic optimization, the final 20 µL multiplex qPCR mixture consisted of: 4 µL of 5× Super Multiple qPCR Premix, 0.4 µL each of the *BclA* and *sap* primers (10 µM), 0.6 µL of the *cap* primer (10 µM), 0.2 µL each of the *BclA* and *sap* TaqMan probes (10 µM), 0.3 µL of the *cap* probe (10 µM), 2 µL of template DNA, and nuclease-free water to volume. Amplification was performed with the following protocol: initial incubation at 37℃ for 120 s, pre-denaturation at 95℃ for 30 s, followed by 45 cycles of denaturation at 95℃ for 5 s and combined annealing/extension at 58℃ for 30 s, with fluorescence acquisition at the end of each annealing/extension step.

### Standard curve of the multiplex qPCR assay

Serial 10-fold dilutions (10^7^ to 10^0^ copies/μL) of the recombinant plasmid standards for the *BclA*, *cap*, and *sap* genes were analyzed using the established multiplex TaqMan qPCR assay. Amplification curves for all targets displayed characteristic sigmoidal shapes across the entire concentration range (Fig. 2). The corresponding standard curves demonstrated excellent linearity, with the following regression equations and correlation coefficients: for *sap* (pMD19-sap), *y* = –3.4455*x* + 38.806, *R*^2^ = 0.996 (Fig. 2A); for *cap* (pMD19-cap), *y* = –3.5656*x* + 38.824, *R*^2^ = 0.9977 (Fig. 2B); and for *BclA* (pMD19-BclA), *y* = –3.5452*x* + 39.05, *R*^2^ = 0.9956 (Fig. 2C).

**Fig 2:**
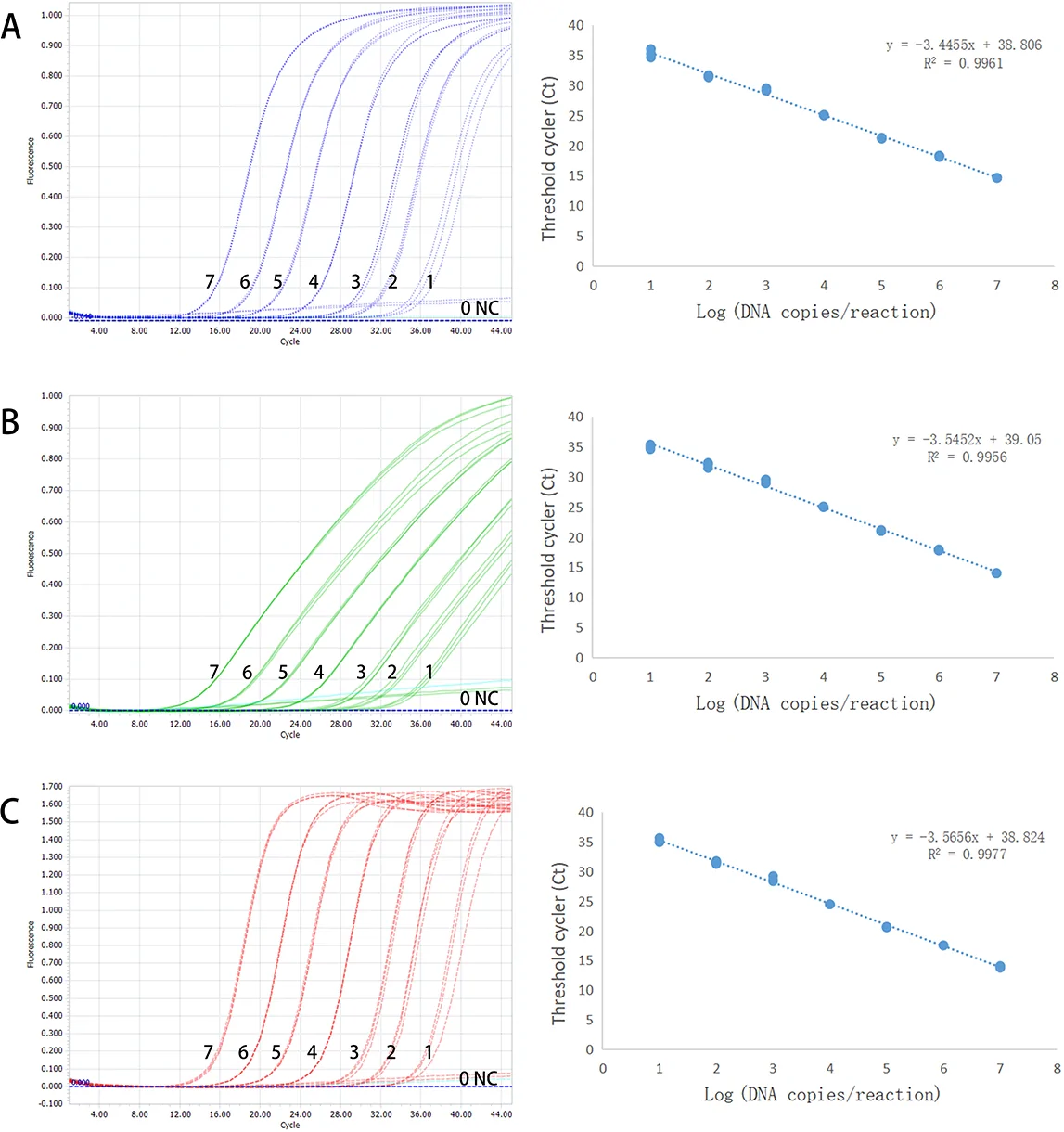
Standard curves and amplification plots of the multiplex qPCR assay. (**A**) *sap* gene (pMD19-sap plasmid), (**B**) *cap* gene (pMD19-cap plasmid), and (**C**) *BclA* gene (pMD19-BclA plasmid). In each panel, amplification plots (left) show fluorescence versus cycle number for 10-fold serial dilutions of plasmid standards (curves 1–7, from 2.11 × 10^7^ to 2.11× 10^0^ copies/μL). The corresponding standard curves (right) plot quantification cycle (Cq) against the logarithm of plasmid copy number. Linear regression equations and correlation coefficients (*R*^2^) are displayed for each target.

### Analytical specificity

The specificity of the multiplex qPCR assay was evaluated using genomic DNA from 11 non-*B*. *anthracis* strains, including other *Bacillus* species and common pathogens. A Cq value of < 35 was defined as a positive detection. Recombinant plasmid controls for all three target genes yielded strong positive signals (mean Cq: *sap*, 21.03; *BclA*, 21.50; *cap*, 20.73). No cross-reactivity was observed for *sap* or *cap* with any of the non-target strains (all Cq values were either ≥36 or undetected, i.e., negative). In contrast, a specific amplification signal for the *BclA* target (Cq = 22.75) was detected exclusively in *Bacillus cereus* (Fig. 3). This distinct amplification profile—*BclA*-positive but *sap*- and *cap*-negative in *B. cereus*—enables clear discrimination between *B. anthracis* and this closely related species, confirming the high specificity of the multiplex assay.

**Fig 3:**
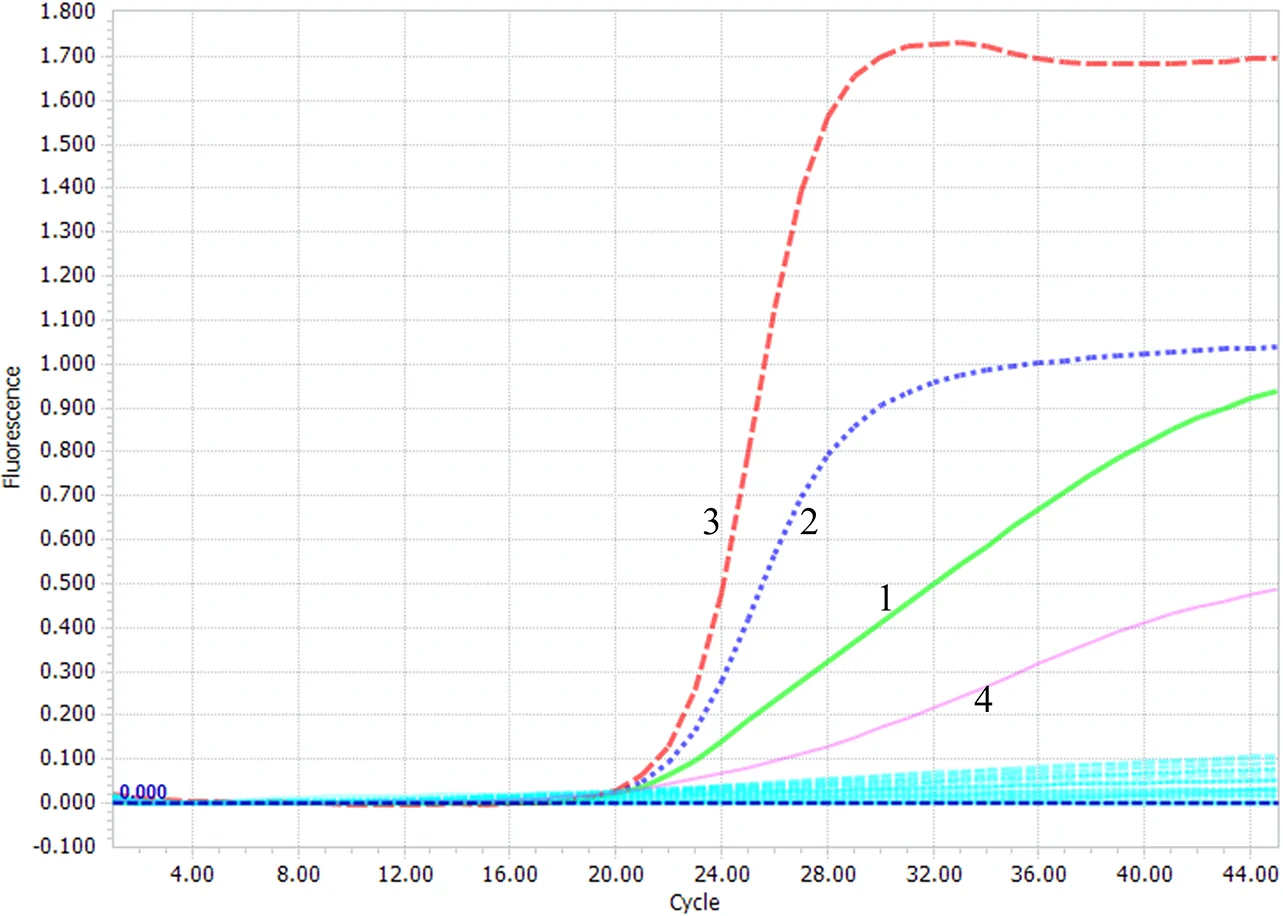
Specificity validation of the multiplex TaqMan qPCR assay. The amplification curves demonstrate the assay’s specificity for each target gene. Curve 1: *BclA* gene (pMD19-BclA plasmid). Curve 2: *sap* gene (pMD19-sap plasmid). Curve 3: *cap* gene (pMD19-cap plasmid). Curve 4: Genomic DNA from *Bacillus cereus*, which cross-reacts only with the *BclA* target probe. No amplification was observed for the *sap* and *cap* genes in any of the other tested non-*B*. *anthracis* bacterial strains (data not shown).

### Combined identification of wild-type and vaccine strains of *B. anthracis*

Genomic DNA from two vaccine strains—the capsule-producing Chinese vaccine strain II (pXO1**^+^**, pXO2**^+^**) and the acapsular Sterne strain 34F2 (pXO1**^+^**, pXO2^−^)—and two wild-type strains (HC1 and SX1) of *B. anthracis* was analyzed using the established multiplex qPCR assay (Cq < 35 defined as positive). Distinct amplification profiles were obtained (Fig. 4A). The Chinese vaccine strain II yielded positive signals for all three targets (*sap*, *cap*, and *BclA*), with Cq values of 21.01 (*BclA*), 17.53 (*sap*), and 17.01 (*cap*), respectively. The acapsular Sterne strain 34F2 was positive only for *sap* (Cq = 13.94), while *cap* and *BclA* were not detected. The wild-type strain HC1 was positive for *sap* and *cap* (Cq = 24.77 and 23.87, respectively) but negative for *BclA*. The wild-type strain SX1 exhibited a profile identical to the Chinese vaccine strain II, with positive amplification for all three targets (Cq: 22.56 for *BclA*, 18.83 for *sap*, and 18.02 for *cap*). All obtained Cq values were well below the positive cutoff of 35.

**Fig 4:**
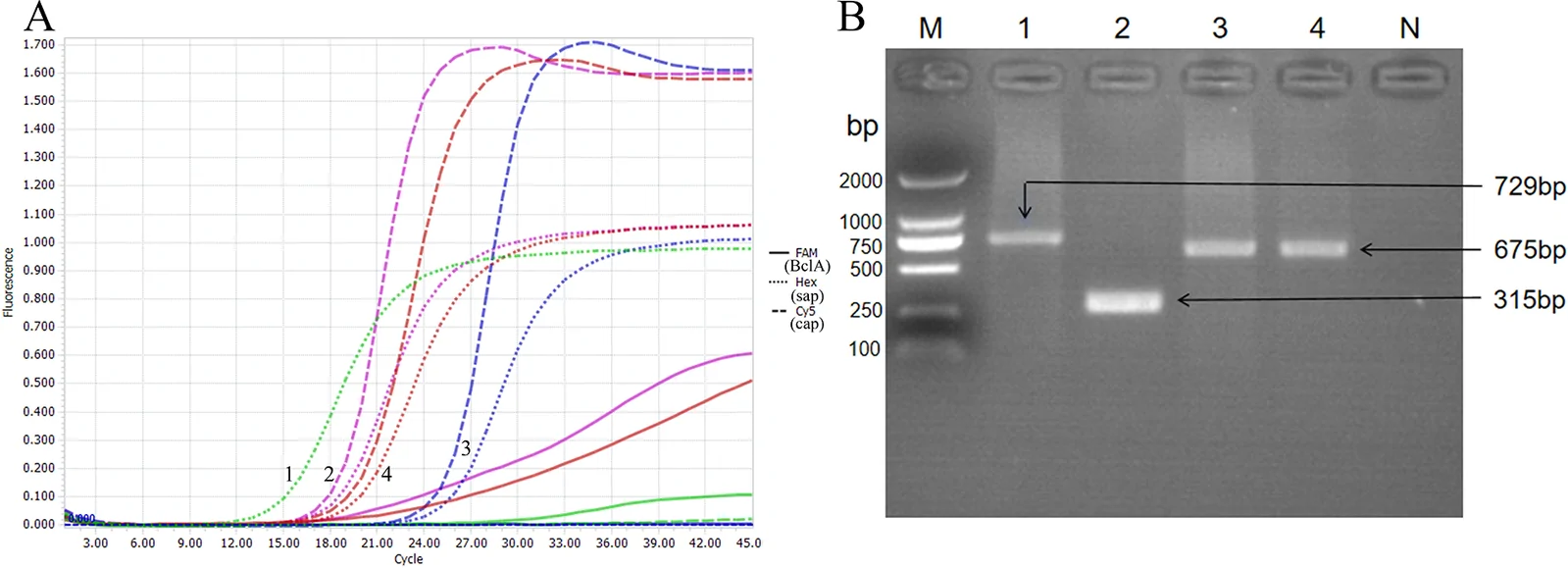
Discrimination of *B. anthracis* wild-type and vaccine strains using multiplex TaqMan qPCR and *BclA* gene length analysis. The figure integrates the results of two complementary assays. (**A**) Amplification profiles from the multiplex qPCR assay for the acapsular Sterne strain 34F2 (green), Chinese vaccine strain II (purple), wild-type strain SX1 (red), and wild-type strain HC1 (blue). (**B**) Agarose gel electrophoresis of conventional PCR products targeting the *BclA* gene. Lane 1: Acapsular Sterne strain 34F2 (*sap^+^/cap^−^/BclA*^−^ in qPCR; ~729 bp amplicon). Lane 2: Chinese vaccine strain II (*sap^+^/cap^+^/BclA ^+^* in qPCR; ~315 bp amplicon). Lane 3: wild-type strain HC1 (*sap^+^/cap^+^/BclA*^−^ in qPCR; ~675 bp amplicon). Lane 4: wild-type strain SX1 (*sap^+^/cap^+^/BclA^+^* in qPCR; ~675 bp amplicon). Lane N: negative control (nuclease-free water). For acapsular Sterne strains 34F2 and HC1, the *BclA* gene was not detected by qPCR (Cq ≥ 35, scored as negative) due to nucleotide mismatches in the probe binding region (confirmed by sequencing), whereas the primer binding sites remain conserved, allowing conventional PCR to produce an amplicon.

To further resolve strains sharing identical qPCR profiles, conventional PCR targeting the *BclA* gene was performed. Electrophoresis revealed distinct amplicon sizes (Fig. 4B): approximately 729 bp for the acapsular Sterne strain 34F2, 315 bp for the Chinese vaccine strain II, and 675 bp for both wild-type strains (HC1 and SX1).

The integration of multiplex qPCR profiles with *BclA* fragment length polymorphism enabled a conclusive identification strategy, as summarized in Table 1. This combined approach allows for clear and reliable discrimination between the vaccine and wild-type strains of *B. anthracis*, successfully resolving even those with closely related qPCR profiles.

**TABLE 1:** Integration of multiplex qPCR profiles with *BclA* fragment length polymorphism enabled a conclusive identification strategy^*a*^

Strain	qPCR profile (*sap* / *cap* / *BclA*)	*BclA* amplicon size	Discriminatory basis
Acapsular Sterne strain 34F2	+/ − / −	~729 bp	Unique qPCR profile
Wild-type HC1	+/ + / −	~675 bp	Distinct qPCR profile (BclA^−^)
Chinese vaccine II	+ / + / +	~315 bp	Shared qPCR profile with SX1, but differentiated by unique BclA amplicon size
Wild-type SX1	+/ + / +	~675 bp	Shared qPCR profile with Chinese vaccine II, but distinguished by BclA amplicon

^*a*^ A Cq value of <35 was defined as positive for qPCR detection. For acapsular Sterne strain 34F2 and wild-type HC1, the negative *BclA* qPCR signal (Cq ≥35) is attributable to nucleotide mismatches in the probe binding region, whereas the primer binding sites remain conserved, allowing conventional PCR amplification (see Fig. 4 legend for details).

### Sensitivity of the multiplex qPCR assay

The sensitivity of the multiplex qPCR assay was evaluated using ten-fold serial dilutions of the standard plasmids (pMD19-sap, pMD19-cap, and pMD19-BclA), ranging from 2.11×10^7^ to 2.11×10 copies/μL. The assay demonstrated high sensitivity for all three targets, with a consistent limit of detection (LOD) of 2.11×10^1^ copies/μL (Cq <35) for each plasmid (Fig. 5). The corresponding Cq values for both singleplex and multiplex qPCR formats are summarized in Table 2, which illustrates comparable performance between the two assay formats across the tested concentration range.

**Fig 5:**
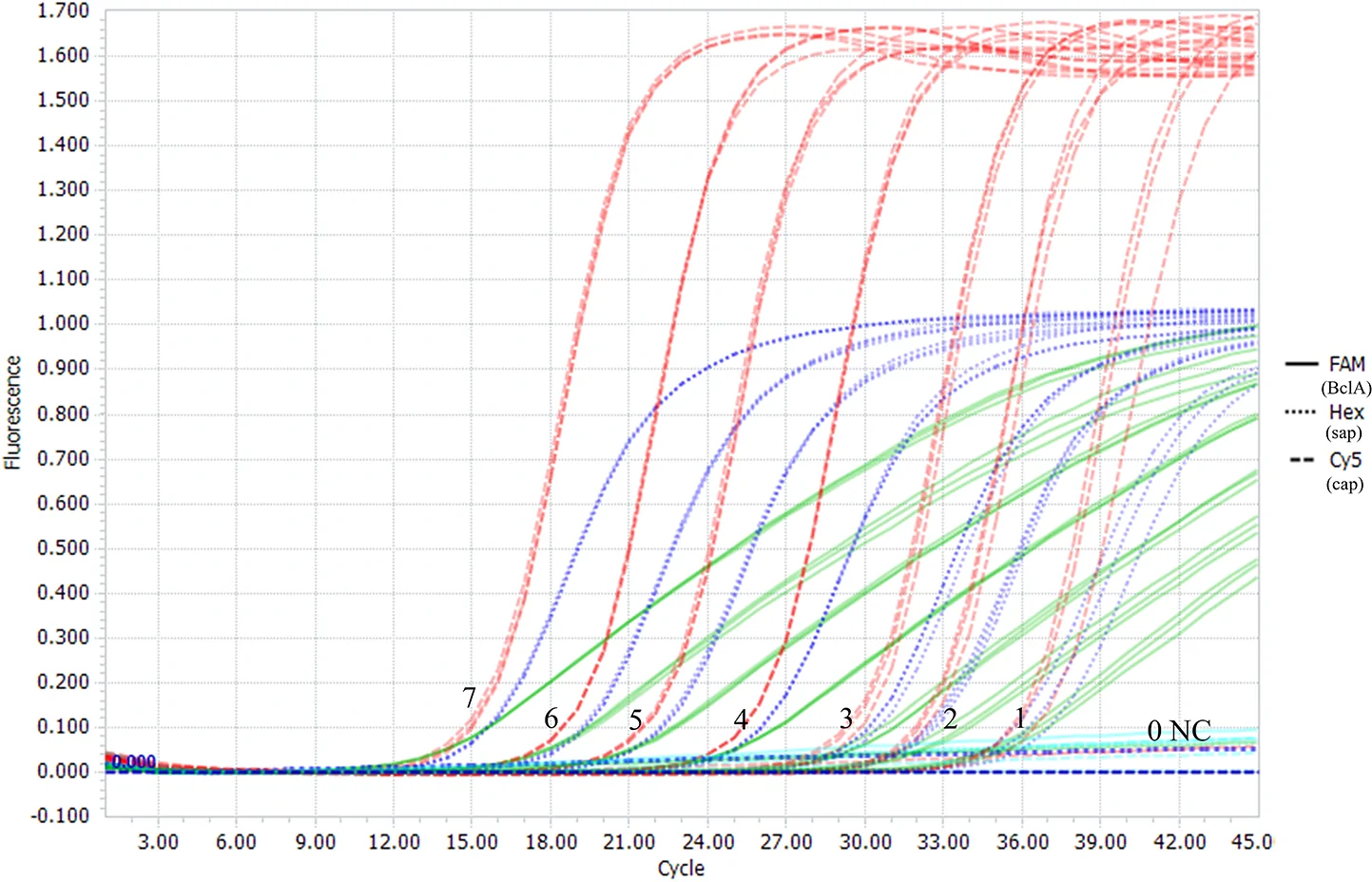
Determination of the limit of detection (LOD) for the multiplex TaqMan qPCR assay. Serial 10-fold dilutions of a mixture containing the pMD19-sap, pMD19-cap, and pMD19-BclA plasmid standards, ranging from 2.11 × 10^0^ to 2.11 × 10^7^ copies/μL, were amplified in the multiplex qPCR assay. Amplification curves for all three targets are shown for each dilution (curves 0–7). The assay consistently detected each target down to 2.11 × 10 copies/μL, with quantification cycle (Cq) values < 35 at this concentration, confirming a uniform LOD for the *sap*, *cap*, and *BclA* genes.

**TABLE 2:** Comparison of Cq values between singleplex and multiplex qPCR assays^*a*^

Target gene	Assay type	Concentration (copies/μL)							
		2.11 × 10^7^	2.11 × 10^6^	2.11 × 10^5^	2.11 × 10^4^	2.11 × 10^3^	2.11 × 10^2^	2.11 × 10^1^	2.11 ×10^0^
*sap*	Singleplex qPCR	14.00	17.07	20.29	23.84	28.11	31.57	35.06	38.28
	Multiplex qPCR	14.64	18.24	21.23	25.04	29.24	31.50	35.28	–
*cap*	Singleplex qPCR	13.36	16.42	19.86	23.28	27.68	31.58	34.79	–
	Multiplex qPCR	13.93	17.54	20.62	24.45	28.69	31.48	35.22	36.96
*BclA*	Singleplex qPCR	13.36	16.96	20.42	23.97	28.44	32.25	35.64	–
	Multiplex qPCR	14.00	17.85	21.05	25.00	29.30	31.93	34.95	–

^*a*^ –, no amplification detected (no Cq value obtained).

### Analytical repeatability

The repeatability of the multiplex qPCR assay was evaluated by testing 10-fold serial dilutions of the standard plasmids mixture (2.11 × 10^7^ to 2.11 × 10^0^ copies/μL) in three independent runs. Intra-assay and inter-assay coefficients of variation (CV) were calculated for each target gene across the concentration series. As summarized in Table 3, the intra-assay CV values were all below 1.96%, and the inter-assay CV values were below 3.06%. These results demonstrate that the established multiplex qPCR assay exhibits high repeatability and reliable performance across repeated experiments.

**TABLE 3:** Intra-group and inter-group repeatability test results

Plasmid standard mixture	Copies/μL	Intra-group repeatability tests		Inter-group repeatability tests	
		Cq (x ± s)	CV/%	Cq (x ± s)	CV/%
pMD19-sap	10^7^	14.64 ± 0.02	0.14%	14.43 ± 0.30	2.10%
	10^6^	18.24 ± 0.11	0.60%	18.00 ± 0.52	2.91%
	10^5^	21.23 ± 0.09	0.40%	20.95 ± 0.42	2.00%
	10^4^	25.04 ± 0.06	0.22%	24.66 ± 0.55	2.24%
	10^3^	29.24 ± 0.22	0.76%	28.93 ± 0.52	1.79%
	10^2^	31.50 ± 0.18	0.57%	31.48 ± 0.16	0.50%
	10^1^	35.28 ± 0.69	1.96%	34.99 ± 0.72	2.06%
pMD19-cap	10^7^	13.93 ± 0.16	1.17%	13.82 ± 0.28	2.06%
	106	17.54 ± 0.02	0.11%	17.36 ± 0.53	3.06%
	10^5^	20.62 ± 0.06	0.30%	20.43 ± 0.33	1.63%
	10^4^	24.45 ± 0.01	0.04%	24.10 ± 0.52	2.17%
	10^3^	28.69 ± 0.46	1.60%	28.44 ± 0.54	1.92%
	10^2^	31.48 ± 0.25	0.79%	31.49 ± 0.18	0.59%
	10^1^	35.22 ± 0.37	1.06%	35.31 ± 0.54	1.53%
pMD19-BclA	10^7^	14.00 ± 0.02	0.12%	13.85 ± 0.28	2.01%
	10^6^	17.85 ± 0.10	0.55%	17.69 ± 0.42	2.35%
	10^5^	21.05 ± 0.10	0.46%	20.88 ± 0.28	1.35%
	10^4^	25.00 ± 0.06	0.23%	24.66 ± 0.49	1.97%
	10^3^	29.30 ± 0.34	1.17%	29.09 ± 0.45	1.53%
	10^2^	31.93 ± 0.43	1.35%	31.93 ± 0.38	1.19%
	10^1^	34.95 ± 0.38	1.09%	35.27 ± 0.53	1.50%

### Validation with wild-type *B. anthracis* strains

The established multiplex TaqMan qPCR assay was applied to analyze genomic DNA from a panel of 19 wild-type *B. anthracis* strains (including HC1 and SX1). Distinct amplification profiles were obtained for these strains (Fig. 6A). Strains HC1, GX5, SL1, SL2, SL3, M9, GQ2, SY1, 2a, ZL2-3, and ZL1-2 were positive for both the *sap* and *cap* targets (Cq <  35) but negative for *BclA*. In contrast, strains SX1, t23, r2, r9, DN3, GZL1, 9a, and ZL2-2 exhibited a profile identical to that of the Chinese vaccine strain II, showing positive amplification for all three targets (Cq <  35).

**Fig 6:**
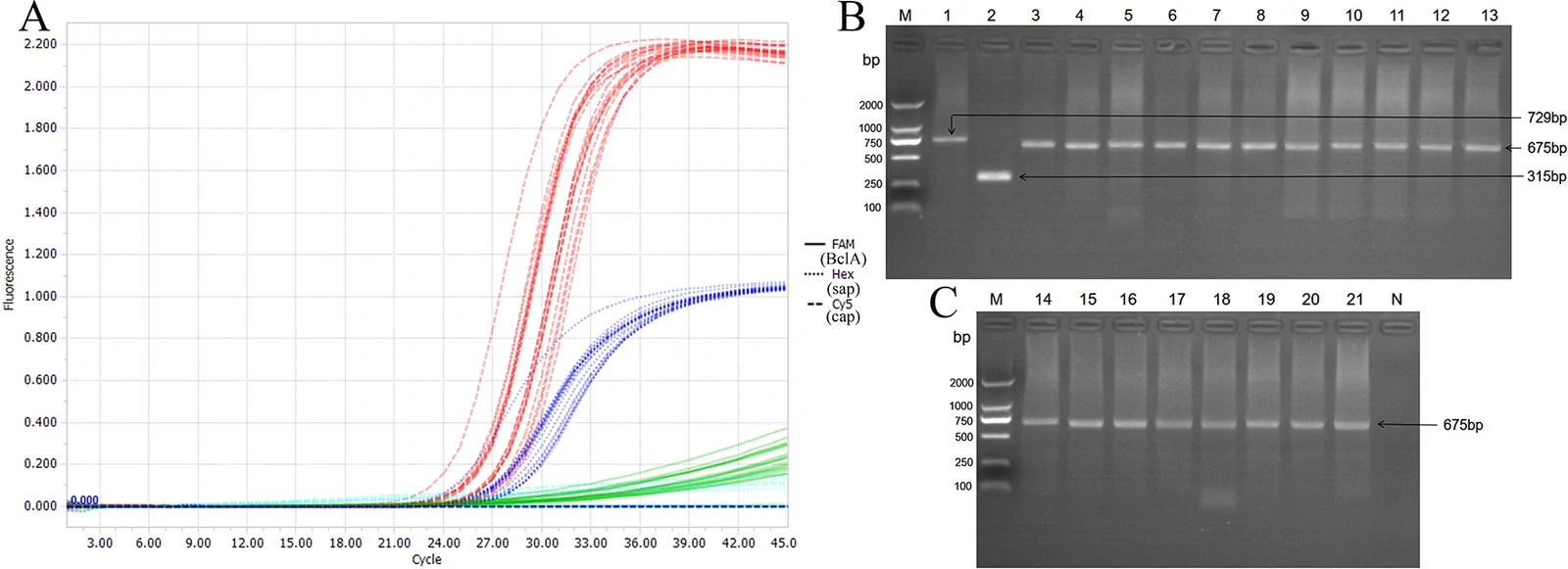
Discrimination of clinical *B. anthracis* isolates using multiplex TaqMan qPCR and BclA gene length analysis. The figure integrates the results of two complementary assays. (**A**) Multiplex qPCR amplification profiles for the *sap*, *cap*, and *BclA* genes. (**B**) Agarose gel electrophoresis of conventional PCR products from the *BclA* gene. Lane 1: acapsular Sterne strain 34F2 (sap^+^/cap^−^/BclA^−^ in qPCR; ~729 bp amplicon). Lane 2: Chinese vaccine strain II (sap^+^/cap^+^/BclA^+^ in qPCR; ~315 bp amplicon). Lanes 3–22: wild-type strains HC1, SX1, GX5, t23, r2, r9, DN3, SL1, SL2, SL3, M9, GQ2, SY1, GZL1, 2a, 9a, ZL2-2, ZL2-3, and ZL1-2 (qPCR: sap^+^/cap^+^/BclA^−^ (BclA+); ~675 bp amplicon). Lane N: negative control (nuclease-free water).

Subsequent conventional PCR analysis of the *BclA* gene revealed consistent fragment length polymorphism (Fig. 6B and C). All 19 wild-type strains yielded a single amplicon of approximately 675 bp, clearly distinguishing them from the acapsular Sterne strain 34F2 (~729 bp) and the Chinese vaccine strain II (~315 bp).

By integrating the multiplex qPCR profile with the *BclA* amplicon size, the confirmatory strategy established previously successfully discriminated the acapsular Sterne strain 34F2, the Chinese vaccine strain II, and all wild-type *B. anthracis* strains within the test panel.

### Comparison of representative global strains using simulated PCR

Using primers from the literature by the World Health Organization (WHO) (1), P. Keim (12), H. Ogawa (13), and T. Zorigt (14), we performed simulated PCR on representative *B. anthracis* strains covering major canSNP genotypes (15, 16) from the National Center for Biotechnology Information (NCBI) database. These methods successfully distinguished wild-type strains from vaccine strains that lack pXO1 or pXO2 plasmids, but they failed to differentiate vaccine strains that carry both virulence plasmids (see Supplemental material for detailed strain information and gel images).

Simulated PCR was performed on the same strain panel using the *BclA* primer pair designed in this study. The results revealed distinct amplification profiles (Tables 4 and 5). Wild-type strains (Ames, Shikan-NIID, Ohio ACB, IAL 52, A0248) and vaccine strains (A16R, Brazilian Vaccinal, acapsular Sterne strain 34F2, V770-NP1-R) produced BclA amplicons ranging from 558 to 729 bp. Wild-type strains (Vollum, HYU01, BA1035, 2000031021) and the Pasteur vaccine strains gave amplicons of 198–297 bp. The *BclA* gene could not be amplified from the Tsiankovskii-I strain. Importantly, all these strains were clearly distinguishable from the Chinese vaccine strain II (315 bp).

**TABLE 4:** Simulated PCR results of wild-type strains^*a*^

Strain designation	This study	WHO (1)			P. Keim (12)			Ogawa H (13)			Zorigt T (14)		
	*BclA*	*sap*	*pag*	*cap*	*VrrC*	*pXO1-AAT*	*pXO2-AT*	*BA_5031*	*pag*	*cap*	*BA5361*	*pag*	*capA*
Ames	675	639	597	847	580	126	141	1,027	364	578	392	612	152
Shikan-NIID	648	639	597	847	580	132	137	1,027	364	578	392	612	152
Ohio ACB	558	639	597	847	580	132	141	1,027	364	578	392	612	152
IAL 52	684	639	597	847	580	132	141	1,027	364	578	392	612	152
A0248	675	639	597	847	580	126	141	1,027	364	578	392	612	152
Canadian bison	315	639	597	847	616	129	135	1,023	364	578	392	612	152
Vollum	225	639	597	847	535	135	139	1,027	364	578	392	612	152
HYU01	288	639	597	847	580	129	137	1,031	364	578	392	612	152
BA1035	297	639	597	847	580	126	143	1,027	364	578	392	612	152
2000031021	252	639	–	847	364	–	132	1,035	–	578	392	–	152

^*a*^ –, no amplification. All numbers are amplicon sizes in base pairs (bp). References: WHO (1), Keim et al. (12), Ogawa et al. (13), and Zorigt et al. (14). The BclA column under “This study” refers to the primer pair designed in this work.

**TABLE 5:** Simulated PCR results of vaccine strains^*a*^

Strain designation	This study	WHO (1)			P. Keim (12)			Ogawa H (13)			Zorigt T (14)		
	*BclA*	*sap*	*pag*	*cap*	*VrrC*	*pXO1-AAT*	*pXO2-AT*	*BA_5031*	*pag*	*cap*	*BA5361*	*pag*	*capA*
A16R	693	639	597	–	580	126	–	1,027	364	–	392	612	–
Brazilian Vaccinal	729	639	597	–	580	132	–	1,027	364	–	392	612	–
Acapsular Sterne strain 34F2	729	639	597	–	580	132	–	1,027	364	–	392	612	–
V770-NP1-R	702	639	597	–	616	132	–	1,027	364	–	392	612	–
Tsiankovskii-I	–	639	597	847	580	126	141	1,027	364	578	392	612	152
Chinese vaccine strain II	315	639	597	847	616	135	137	1,027	364	578	392	612	152
STI1	315	639	–	–	616	–	–	1,027	–	–	392	–	–
Pasteur	198	639	–	847	616	–	137	1,023	–	578	392	–	152
Carbosap	315	639	597	847	616	129	137	1,027	364	578	392	612	152

^*a*^ –, no amplification. All numbers are amplicon sizes in base pairs (bp). References: WHO (1), Keim et al. (12), Ogawa et al. (13), and Zorigt et al. (14). The BclA column under “This study” refers to the primer pair designed in this work.

However, wild-type Canadian bison and vaccine strains STI1 and Carbosap also yielded a 315 bp *BclA* amplicon identical to that of Chinese vaccine strain II. Among these, STI1 is pXO2^−^ and can be differentiated by its cap^−^-negative qPCR profile (sap^+^/cap^−^/BclA^+^), whereas Carbosap and Canadian bison are pXO2^+^ and share the same qPCR profile (sap^+^/cap^+^/BclA^+^) as Chinese vaccine strain II. Therefore, our assay cannot distinguish Carbosap or Canadian bison from Chinese vaccine strain II. Nevertheless, these genotypes are extremely rare in China. In contrast, the vast majority of *B. anthracis* field isolates in China belong to A.Br.Ames and A.Br.001/002 canSNP subgroups, both of which produce a characteristic 675 bp *BclA* amplicon. This size is clearly distinct from the 315 bp amplicon of the Chinese vaccine strain II and the 729 bp amplicon of the acapsular Sterne strain. Therefore, within the Chinese epidemiological context, our method reliably discriminates wild-type from vaccine strains.

## DISCUSSION

The accurate differentiation between wild-type and vaccine strains of *B. anthracis* is a critical unmet need in anthrax surveillance and outbreak management. This is particularly vital in regions using live-spore veterinary vaccines, such as the widely used Chinese vaccine strain II in China. The environmental persistence of spores, whether from wild-type or vaccine strains, complicates source attribution during investigations. Therefore, diagnostics capable of precise strain discrimination are essential for effective epidemiological response and risk assessment.

Current molecular diagnostics for *B. anthracis*, including SYBR Green and TaqMan probe-based qPCR assays, offer high sensitivity but cannot differentiate vaccine from wild-type strains (10, 17, 18). Isothermal amplification methods like LAMP, while promising for field use, share this limitation (19, 20). Strategies targeting genetic polymorphisms, such as variations in the *BclA* gene (4), have been explored. Conventional PCR assays based on *BclA* length can differentiate strains (21, 22) but suffer from lower sensitivity, often requiring culture enrichment that delays results.

In the late 1990s and early 2000s, pioneering studies by P. Keim and colleagues (12) developed multiple-locus variable-number tandem repeat analysis (MLVA) for *B. anthracis*, which could discriminate between vaccine and wild-type strains but required post-PCR fragment sizing using automated DNA sequencers. Subsequently, canonical single-nucleotide polymorphism (canSNP) genotyping (15, 16) provided higher phylogenetic resolution but typically requires multiple separate reactions or specialized equipment.

To systematically evaluate the limitations of existing PCR-based methods, we performed simulated PCR using primer sets reported by WHO (1), Keim et al. (12), Ogawa et al. (13), and Zorigt et al. (14) on 19 publicly available *B. anthracis* genomes representing major canSNP genotypes (15, 16) from the NCBI database. These methods distinguished wild-type strains from vaccine strains lacking pXO1 or pXO2, but they failed to differentiate vaccine strains that carry both virulence plasmids (e.g., the Chinese vaccine strain II) from certain wild-type strains.

When we applied our *BclA* primer pair to the same strain panel, Chinese vaccine strain II yielded a 315 bp amplicon, and this same fragment size was also observed in some wild-type (Canadian bison) and vaccine strains (STI1 and Carbosap). Among these, STI1 is pXO2⁻ and gives a cap^−^-negative qPCR profile (sap^+^/cap^−^/BclA^+^), which readily distinguishes it from Chinese vaccine strain II (sap^+^/cap^+^/BclA^+^). However, Canadian bison (wild-type, pXO2^+^) and Carbosap (vaccine strain, pXO2^+^) share both the qPCR profile (sap^+^/cap^+^/BclA^+^) and the 315 bp BclA fragment with Chinese vaccine strain II, and our current markers cannot differentiate these strains from the Chinese vaccine strain II. These rare genotypes are not endemic to China and, therefore, do not compromise the practical utility of the assay. In contrast, the vast majority of *B. anthracis* field isolates in China belong to the dominant A.Br.001/002 lineage or its closely related A.Br.Ames sub-lineage, which produce a characteristic 675 bp BclA amplicon clearly distinct from the 315 bp fragment of the Chinese vaccine strain II (23–25). Thus, within the Chinese epidemiological context, our integrated strategy offers a decisive advantage over earlier methods: it reliably discriminates wild-type from vaccine strains, including those that carry both virulence plasmids, using a two-step workflow that combines multiplex qPCR with conventional PCR and gel electrophoresis.

These results demonstrate that our two-step strategy successfully resolves the key limitation of earlier methods that failed to differentiate vaccine strains carrying both virulence plasmids. By merging the speed and sensitivity of real-time PCR with the resolving power of a stable genetic marker, this approach provides a clear, practical workflow for discriminating wild-type from vaccine strains, particularly within the Chinese epidemiological context where the vast majority of field isolates produce a 675 bp BclA amplicon. This offers a practical solution to a longstanding diagnostic challenge in anthrax epidemiology and control.

## MATERIALS AND METHODS

### Bacterial strains

The *B. anthracis* strains used in this study were obtained from the culture collection of the Changchun Veterinary Research Institute, Chinese Academy of Agricultural Sciences. They comprised two attenuated vaccine strains and a panel of 19 wild-type isolates. The vaccine strains included the Chinese vaccine strain II (CVCC40202, pXO1^+^, pXO2^+^) and the acapsular Sterne strain 34F2 (CVCC40205, pXO1^+^, pXO2^−^). The wild-type strains (HC1, SX1, GX5, t23, r2, r9, DN3, SL1, SL2, SL3, M9, GQ2, SY1, GZL1, 2a, 9a, ZL2-2, ZL2-3, ZL1-2), which were isolated from various hosts and locations in China between 2009 and 2021 (see Table 6 for details), were employed for validation of the strain discrimination assay.

**TABLE 6:** Information on *Bacillus anthracis* wild-type strains used in this study

Strain designation	Collection year	Location (province)	Source
HC1	2011	Liaoning	Cattle
SX1	2021	Shanxi	Cattle
GX5	2016	Guangxi	Horse
t23	2016	Guangxi	Soil
r2	2016	Guangxi	Horse
r9	2016	Guangxi	Horse
DN3	2016	Jilin	Cattle
SL1	2018	Jilin	Cattle
SL2	2018	Jilin	Cattle
SL3	2018	Jilin	Cattle
M9	2018	Inner Mongolia	Cattle
GQ2	2015	Shanxi	Donkey
SY1	2009	Jilin	Cattle
GZL1	2010	Jilin	Cattle
2a	2012	Liaoning	Cattle
9a	2012	Liaoning	Cattle
ZL2-2	2014	Jilin	Sheep
ZL2-3	2014	Jilin	Sheep
ZL1-2	2012	Jilin	Cattle

Non-target strains for specificity testing included *B. cereus*, *B. thuringiensis*, *B. subtilis*, *Bacillus* sp*.*, *Salmonella* spp., *Streptococcus pasteurianus*, *Pseudomonas aeruginosa*, *Escherichia coli*, *Proteus mirabilis*, *Staphylococcus aureus*, and *Enterococcus faecalis*, all were obtained from and preserved at the New Veterinary Drug Development Laboratory (NDDL) at Shihezi University.

### DNA extraction

Genomic DNA was extracted from all bacterial strains using a standardized protocol. Briefly, overnight cultures were prepared by incubating bacteria in Brain Heart Infusion (BHI) broth at 37°C with shaking at 180 rpm for 18 h. A 3 mL aliquot of each culture was centrifuged (16,760 × *g*, 5 min, 4°C). The pellet was washed and resuspended in 1 mL of sterile deionized water. To ensure efficient lysis, particularly for spore-forming and gram-positive bacteria, 250 mg of acid-washed glass beads (0.1 mm diameter, Sigma-Aldrich) and 500 µL of lysis buffer were added to the suspension, followed by mechanical disruption via vortexing at 1,800 rpm for 30 min. Genomic DNA was then purified from the lysate using the QIAamp DNA Microbiome Kit (QIAGEN, Valencia, CA, USA) according to the manufacturer’s instructions. The eluted DNA was quantified, aliquoted, and stored at –20°C until use.

### Design of primers and TaqMan probes for multiplex qPCR

Primers and corresponding TaqMan probes targeting the conserved regions of the *sap*, *BclA*, and *cap* genes were designed using Primer Premier 5 software. The design was based on the complete genome sequences of the *B. anthracis* Ames Ancestor (GenBank accession no. GCF_000008445.1) and the Chinese vaccine strain II (GCF_022014755.1) to ensure broad applicability. The probes were labeled with distinct fluorophores and a common quencher: FAM-BHQ1 for *BclA*, HEX-BHQ1 for *sap*, and Cy5-BHQ1 for *cap* (Table 7). The specificity and conservation of the designed oligonucleotides were confirmed through *in silico* analysis against the NCBI nucleotide database using the BLAST tool and by alignment with published *B. anthracis* sequences.

**TABLE 7:** Oligonucleotide primers and probes used in this study

Target gene	Oligonucleotide name	Sequence description	Amplicon size (bp)
*sap*	sap-F	TGATGAGCCAACAGCATTACA	93
	sap-R	GCAGCTGGCGTTACAAATTC	
	sap-Probe	CGGTACTGAAGTTGTTTCACCAGAGGG	
*cap*	cap-F	TTACTGGTGCTACTGCTTCTG	91
	cap-R	CCTCGTTATGTAGCAATCGTAT	
	cap-Probe	ACGTTGTACCCATGTCGCAGCTATTAA	
*BclA*	BclA-F	AGTCCTGCTGGAAGTCCTAGTC	315
	BclA-R	ACCACCGATACCACCATTTACC	
	BclA-Probe	TCGGTCCAGTCAGTCCGGTAGCACC	

### Construction of plasmid standards

The *sap*, *BclA*, and *cap* genes were individually amplified by PCR using genomic DNA from the *B. anthracis* Chinese vaccine strain II as the template and the primers listed in Table 4. The amplification products were separated by 1% agarose gel electrophoresis, and the target bands were purified using a Gel Extraction Kit (Tiangen Biotech, Beijing, China). The purified fragments were ligated into the pMD19-T vector (Takara Bio Inc., Dalian, China) and transformed into *E. coli* DH5α competent cells (Takara Bio, Inc., Dalian, China) by heat shock. Positive clones identified by PCR were cultured, and the recombinant plasmids were extracted using an EasyPure EndoFree Plasmid MaxiPrep Kit (TransGen Biotech, Beijing, China). The resulting plasmids, designated pMD19-sap, pMD19-BclA, and pMD19-cap, were stored at –80°C.

Plasmid concentrations were measured using a NanoDrop 2000 spectrophotometer (Thermo Fisher Scientific, USA). Copy numbers were calculated using the following formula: copy number (copies/μL) = [6.02 × 10^23^ × (concentration in ng/μL) × 10^−9^] / [plasmid length (bp) × 660].

### Optimization of singleplex qPCR conditions

Reaction conditions were optimized using standard plasmids at 10^5^ copies/μL as the template by systematically varying the concentrations of primers and probes (final concentration tested: 0.2, 0.4, 0.6, and 0.8 µM) and the annealing temperature (tested at 56, 57, 58, 59, and 60°C). The final optimized 20 µL reaction mixture consisted of the following: 4 µL of 5× BioSmart U + Super Multiple Probe qPCR PreMix (Vazyme Biotech Co., Ltd, Nanjing, China), forward and reverse primers (0.4 µM each), TaqMan probe (0.2 µM), 2 µL of template DNA, and nuclease-free water to volume.

Amplification was performed on a LightCycler 96 System (Roche Life Science, Basel, Switzerland) with the following protocol: initial incubation at 37°C for 120 s, pre-denaturation at 95°C for 30 s, followed by 45 cycles of denaturation at 95°C for 5 s and combined annealing/extension at 57°C for 30 s, with fluorescence acquisition at the end of each annealing/extension step.

### Optimization of multiplex qPCR conditions

The multiplex qPCR assay was optimized for the simultaneous co-amplification of the *sap*, *cap*, and *BclA* genes. To ensure balanced amplification and minimize inter-channel crosstalk, the primer and probe concentrations were further optimized beyond the singleplex conditions. This was done using an equimolar mixture of the three plasmid standards (pMD19-sap, pMD19-cap, and pMD19-BclA) as template by testing various concentration combinations.

A final reaction formulation was established based on this process. The thermal cycling protocol was identical to that used for the singleplex qPCR. All reactions were run on a LightCycler 96 System, with fluorescence acquisition in the FAM, HEX, and Cy5 channels.

For data analysis, the quantification cycle (Cq) for each target was automatically determined by the LightCycler 96 software (version 1.1) using the second derivative maximum method, with the fluorescence threshold manually set to intersect the linear exponential phase of all standard curve amplifications and consistently positioned above any background signal from non-template controls.

### Specificity of the multiplex qPCR assay

The specificity of the assay was evaluated using genomic DNA from the following 11 non-target bacterial strains: *B. cereus*, *B. thuringiensis*, *B. subtilis*, *Bacillus* sp., *Escherichia coli*, *Proteus mirabilis*, *Pseudomonas aeruginosa*, *Salmonella* spp., *Staphylococcus aureus*, *Enterococcus faecium*, and *Streptococcus pasteurianus*.

### Strain discrimination assay

To validate the strain-discriminatory capability of the assay, total DNA from key *B. anthracis* strains was analyzed. This included a wild-type strain, the acapsular Sterne strain 34F2 (pXO1^+^, pXO2^−^), and the Chinese vaccine strain II (pXO1^+^, pXO2^+^). Each sample was first tested with the established multiplex qPCR assay.

Subsequently, conventional PCR was performed to amplify the *BclA* gene for length polymorphism analysis. The 20 μL PCR mixture contained 10.0 μL of 2 × Taq PCR MasterMix, 0.5 μM each of primers BclA-F and BclA-R (Table 4), and 2 μL of template DNA. Amplification was carried out under the following conditions: 95°C for 5 min; 35 cycles of 95°C for 30 s, 58°C for 30 s, and 72°C for 30 s; and with a final extension at 72°C for 5 min. The resulting amplicons were separated on a 1.0% agarose gel to determine their fragment sizes.

### Sensitivity of the multiplex qPCR assay

The limit of detection (LOD) of the multiplex qPCR assay was determined using 10-fold serial dilutions of the individual standard plasmids (pMD19-sap, pMD19-cap, and pMD19-BclA). Each plasmid was diluted in nuclease-free water across a concentration range from 2.11 × 10^0^ to 2.11 × 10^7^ copies/μL. Each dilution was tested in triplicate. A cycle threshold (Cq) value of <35 was pre-defined as a positive detection event based on established qPCR guidelines for reliable amplification. The LOD was accordingly defined as the lowest concentration at which all three technical replicates yielded a positive signal.

### Repeatability of the multiplex qPCR assay

The repeatability of the multiplex qPCR assay was assessed using 10-fold serial dilutions of a mixture containing the three standard plasmids (pMD19-sap, pMD19-cap, and pMD19-BclA). The dilution series ranged from 2.11 × 10^7^ to 2.11 × 10^1^ copies/μL, and each concentration was tested in triplicate within the same run (intra-assay) and across three independent runs performed on different days (inter-assay). For each target gene, the mean Cq value and standard deviation were calculated at every concentration level. Intra-assay and inter-assay coefficients of variation (CV) were then derived from these values to evaluate the precision of the assay.

### Clinical sample analysis

Total DNA extracted from the 19 wild-type *B. anthracis* strains was analyzed using the established multiplex qPCR assay under the optimized conditions described in the section “Optimization of Multiplex qPCR Conditions.” A mixture of the three standard plasmids at a concentration of 10^4^ copies/μL served as a positive control in each run. Following qPCR analysis, conventional PCR targeting the BclA gene was performed on all samples to assess fragment length polymorphism for definitive strain discrimination.

### Simulated PCR analysis of representative global strains

To evaluate the ability of our assay to discriminate between wild-type and vaccine strains across diverse genetic backgrounds, we performed *in silico* PCR using publicly available *B. anthracis* genomes. Representative strains covering major canSNP genotypes were downloaded from NCBI (15, 16), including vaccine (A16R, Brazilian Vaccinal, acapsular Sterne strain 34F2, V770-NP1-R, Chinese vaccine strain II, Tsiankovskii-I, STI1, Pasteur, and Carbosap) and wild-type (Ames, Shikan-NIID, Ohio ACB, IAL 52, A0248, Canadian bison, Vollum, HYU01, BA1035, and 2000031021) strains (Table 8). Simulated PCR was conducted using primers reported by WHO (1), P. Keim (12), H. Ogawa (13), and T. Zorigt (14), as well as *BclA* primers designed in this study, to evaluate the discriminatory power of the assay.

**TABLE 8:** Representative *Bacillus anthracis* genome assemblies used for *in silico* validation of the multiplex qPCR assay

Strain designation	RefSeq accession	canSNP subgroup	Source
Ames	GCF_000833065.1	A.Br.Ames	America
A16R	GCF_000512775.2	A.Br.001/002	China
Shikan-NIID	GCF_002356575.1	A.Br.001/002	Japan
Brazilian Vaccinal	GCF_002007035.1	A.Br.001/002	Brazil
acapsular Sterne strain 34F2	GCF_024396795.1	A.Br.001/002	China
Ohio ACB	GCF_000832505.1	A.Br.003/004	America
IAL 52	GCF_042193355.1	A.Br.003/004	Brazil
A0248	GCF_000009165.1	A.Br.005/006	Germany
V770-NP1-R	GCF_000832785.1	A.Br.064 (V770)	America
Tsiankovskii-I	GCF_000181675.2	A.Br.008/011	Soviet/Russian
Chinese vaccine strain II	GCF_024396775.1	A.Br.008/011	China
STI1	GCF_002980615.3	A.Br.008/009	Russia
Canadian bison	GCF_000833125.1	A.Br.008/009	Canadian, bison
Pasteur	GCF_000832585.1	A.Br.008/009	United Kingdom
Carbosap	GCF_000310045.1	A.Br.008/009	Italy
Vollum	GCF_000742895.1	A.Br.Vollum	America
HYU01	GCF_000725325.1	B.Br.001/002	South Korea
BA1035	GCF_000832725.1	B.Br.001/002	South Africa
2000031021	GCF_000742655.1	C.Br.001	America

## Supplementary Material

Reviewer comments

## Data Availability

The data sets used and/or analyzed during the current study are available from the corresponding author upon reasonable request.

## SUPPLEMENTAL MATERIAL

Table S1, Fig. S1 to S13 spectrum.00192-26-s0001.docx Information of the simulated PCR.

OPEN PEER REVIEW reviewer-comments.pdf An accounting of the reviewer comments and feedback.

ASM does not own the copyrights to Supplemental Material that may be linked to, or accessed through, an article. The authors have granted ASM a non-exclusive, world-wide license to publish the Supplemental Material files. Please contact the corresponding author directly for reuse.
