# Supplementary material for: An integrated strategy of multiplex qPCR and BclA gene length polymorphism analysis for differentiating Bacillus anthracis wild-type and vaccine strains
Source: Microbiol Spectr. 2026 Sep 1;14(10):e00192-26. doi: 10.1128/spectrum.00192-26 (PMC13688997; doi:10.1128/spectrum.00192-26)
Supplement: Supplementary file 1 [file reviewer-comments.pdf]

ASM | Microbiology Spectrum

An Integrated Strategy of Multiplex qPCR and BclA Gene Length Polymorphism Analysis for Differentiating Bacillus anthracis Wild-Type and Vaccine Strains

Jiaqi Yang, Yan Liang, Bing Liang, Yanfang Li, Lingwei Zhu, and Yonggang Qu

Corresponding Author(s): Yonggang Qu, Shihezi University

Review Timeline:

Submission Date:	January 15, 2026
Editorial Decision:	April 6, 2026
Revision Received:	June 16, 2026
Accepted:	July 24, 2026

Editor: Cecilia Thompson

Reviewer(s): Disclosure of reviewer identity is with reference to reviewer comments included in decision letter(s). The following individuals involved in review of your submission have agreed to reveal their identity: Dongshu Wang (Reviewer #2)

Transaction Report:

(Note: With the exception of the correction of typographical or spelling errors that could be a source of ambiguity, letters and reports are not edited. The original formatting of letters and referee reports may not be reflected in this compilation.)

DOI: https://doi.org/10.1128/spectrum.00192-26

Re: Spectrum00192-26 (An Integrated Strategy of Multiplex qPCR and BclA Gene Length Polymorphism Analysis for Differentiating Bacillus anthracis Wild-Type and Vaccine Strains)

Dear Prof. Yonggang Qu:

Thank you for the privilege of reviewing your work. Below you will find my comments, instructions from the Spectrum editorial office, and the reviewer comments.

For full disclosure, this editor served as the third reviewer for this manuscript. Please see reviewer #3 comments below.

Please return the manuscript within 60 days; if you cannot complete the modification within this time period, please contact me. If you do not wish to modify the manuscript and prefer to submit it to another journal, notify me immediately so that the manuscript may be formally withdrawn from consideration by Spectrum.

Revision Guidelines
To submit your modified manuscript, log into the submission site at https://spectrum.msubmit.net/cgi-bin/main.plex. Go to Author Tasks and click the appropriate manuscript title to begin. The information you entered when you first submitted the paper will be displayed; update this as necessary. Note the following requirements:

• Upload point-by-point responses to the issues raised by the reviewers in a file named "Response to Reviewers," NOT in your cover letter.
• Upload a compare copy of the manuscript (without figures) as a "Marked-Up Manuscript" file.
• Upload a clean .DOC/.DOCX version of the revised manuscript and remove the previous version.
• Each figure must be uploaded as a separate, editable, high-resolution file (TIFF or EPS preferred), and any multipanel figures must be assembled into one file.
• Any supplemental material intended for posting by ASM should be uploaded with their legends separate from the main manuscript. You can combine all supplemental material into one file (preferred) or split it into a maximum of 10 files with all associated legends included.

For complete guidelines on revision requirements, see our Submission and Review Process webpage. Submission of a paper that does not conform to guidelines may delay acceptance of your manuscript.

Data availability: ASM policy requires that data be available to the public upon online posting of the article, so please verify all links to sequence records, if present, and make sure that each number retrieves the full record of the data. If a new accession number is not linked or a link is broken, provide Spectrum production staff with the correct URL for the record. If the accession numbers for new data are not publicly accessible before the expected online posting of the article, publication may be delayed; please contact production staff (Spectrum@asmusa.org) immediately with the expected release date.

Publication Fees: For information on publication fees and which article types are subject to charges, visit our website. If your manuscript is accepted for publication and any fees apply, you will be contacted separately about payment during the production process; please follow the instructions in that e-mail. Arrangements for payment must be made before your article is published.

ASM Membership: Corresponding authors may join or renew ASM membership to obtain discounts on publication fees. Need to upgrade your membership level? Please contact Customer Service at Service@asmusa.org.

The ASM Journals program strives for constant improvement in our submission and publication process. Please tell us how we can improve your experience by taking this quick Author Survey.

Thank you for submitting your paper to Spectrum.

Sincerely,
Cecilia Thompson
Editor
Microbiology Spectrum

Reviewer #2 (Comments for the Author):

The integrated molecular strategy developed in this study provides a reliable, sensitive, and specific tool for precise detection and differentiation of Bacillus anthracis strains, which could significantly enhance both public health and veterinary defense capabilities. However, the following critical issues should be addressed to strengthen the manuscript:
Major Comments：

The primary issue that should be emphasized in the manuscript is to expand the scope of validation by adding more wild-type and vaccine strains to further verify the universality of this strategy. I am particularly concerned about whether the vaccine strain can be distinguished from its parental strain. The manuscript selected CVCC40202 and the Sterne 34F2, which is clearly insufficient. There are sufficient wild-type strains with adequate temporal and geographical coverage, but it remains uncertain whether the genotypes of these strains belong to the same CanSNP subgroup. I understand that strains collection were limited and challenging. It is helpful to analyze genomic information from public databases and incorporate more strains for simulated PCR validation. Please add bioinformatics approaches to address strain limitations (e.g.
STI1，A16R，Carbosap，95014，Shikan-NIID，Brazilian Vaccinal and Pasteur strains） and perform in silico analysis using publicly available B. anthracis genomes .

minor comments：
1.Why is standard plasmid used for the sensitivity testing of Multiplex qPCR Assay instead of Bacillus anthracis genomic DNA?
2.Line 252 and Line 187 in Fig 4，Fig 6, Chinese vaccine strain II CVCC40202 is not Sterne-based vaccine strain.Sterne-type vaccine strains belonged to
A.Br.001/002 subgroup while the CVCC40202 strain belonged to A.Br.008/009 subgroup.

Reviewer #3 (Comments for the Author):

In this manuscript, Yang et. al. describe the performance characteristics of a multiplex PCR assay designed to differentiate wild-type and vaccine strains of Bacillus anthracis. This proof-of-concept work explored specificity, sensitivity, repeatability, and the ability of the multiplex assay to differentiate between wild-type and vaccine strains. To confirm differentiation of vaccine strains, an analysis of the BclA amplicon length was applied. The manuscript is well-written, and the conclusions are scientifically sound. I hope the authors view these suggestions as being constructive and designed to help strengthen their manuscript.

Major Comments
The validation section could be strengthened by an analysis of additional vaccine strains. Only two vaccine strains were included in the technical validation, and it would be beneficial to include data of how other vaccine strains might be identified by this two-step algorithmic approach. Additional analysis could be limited to in-silico analysis if strains are unavailable.
The discussion could be enhanced by a robust review of previous work and where historical methods to differentiate have failed and how this assay in particular builds upon previous work or fulfills a regional gap in B. anthracis diagnostics. Please review literature on the topics covered in your manuscript published in the early 2000s. In particular, review manuscripts authored/coauthored by P Keim, et al, and L.B. Price, et al. highlight assays that differentiated between vaccine and virulent strains that were developed in the 1990s.

Minor Comments
Page 2, Lines 43-45: Sentence is not complete and needs revision.
Page 2, line 47: Please clarify that pXO2, encoding the poly-D-glutamate, includes the cap gene of interest described here.
Page 2, Lines 52-54: Recommend adding a citation to support statement of existing gap in diagnostics.
Page 3, lines 89-96: This section could be moved in its entirety to the methods section.
Figure 4, Table 1: The authors should comment on why the multiplex detecting BclA was negative for Sterne (34F2) and wild-type HC1, but the conventional PCR using the same primers for BclA resulted in a BclA amplicon.
Page 9, line 268: Is this assay intended to be used on cultured isolates or clinical materials (e.g., blood samples from infected individuals)? The authors could expand on the intended applications of this assay and how this diagnostic approach would not require culture enrichment.

Response to Reviewer #2

We sincerely thank you for your positive assessment of our work and for your valuable comments. We have carefully addressed each point, and the manuscript has been substantially improved. Below is our point-by-point response. All changes are highlighted in the revised manuscript.

Major Comments

The primary issue that should be emphasized in the manuscript is to expand the scope of validation by adding more wild-type and vaccine strains to further verify the universality of this strategy. I am particularly concerned about whether the vaccine strain can be distinguished from its parental strain. The manuscript selected CVCC40202 and the Sterne 34F2, which is clearly insufficient. There are sufficient wild-type strains with adequate temporal and geographical coverage, but it remains uncertain whether the genotypes of these strains belong to the same CanSNP subgroup. I understand that strains collection were limited and challenging. It is helpful to analyze genomic information from public databases and incorporate more strains for simulated PCR validation. Please add bioinformatics approaches to address strain limitations (e.g. STI1，A16R，Carbosap，95014，Shikan-NIID，Brazilian Vaccinal and Pasteur strains） and perform in silico analysis using publicly available *B. anthracis* genomes.

Response：Thank you for your careful review and for acknowledging the value of our work. We fully agree that validation should be extended to more strains to verify universality. Since physical access to additional vaccine strains is limited, we have performed a comprehensive in silico analysis using publicly available *B. anthracis* genomes from the NCBI database.

Methodology of in silico validation (added to the manuscript):

We downloaded chromosome and plasmid sequences of 19 representative strains covering major canSNP subgroups, including the requested vaccine strains (STI1, A16R, Carbosap, Shikan-NIID, Brazilian Vaccinal, Pasteur, CVCC40205, V770-NP1-R, Tsiankovskii-I) and wild-type strains (Ames, Ohio ACB, IAL 52, A0248, Canadian bison, Vollum, HYU01, BA1035, 2000031021). Virtual PCR was performed using the primer and probe sequences

listed in Table 7, and the *BclA* amplicon length was calculated directly from the genome sequences. The simulated PCR gel images are provided in the Supplementary Material (Supplementary Table S1 and Supplementary Figures S1-S13).

Key findings from the in silico analysis:

1. The *sap* and *cap* primer/probe binding sites showed no mismatches in any of the 19 tested genomes, indicating high conservation.

2. The *BclA* amplicon length was 315 bp for the Chinese vaccine strain II (CVCC40202) and also for wild-type strains (Canadian bison) and vaccine strains (STI1, Carbosap).

3. Strains that can be distinguished from Chinese vaccine strain II (CVCC40202):

STI1 are $pXO2^{-}$ (acapsular), giving a *cap*-negative qPCR profile ($sap^{+}/cap^{-}/BclA^{+}$), whereas CVCC40202 is $sap^{+}/cap^{+}/BclA^{+}$. Therefore, they are readily differentiated by the multiplex qPCR alone, even though their *BclA* fragment length is identical.

4. Strains that cannot be distinguished from Chinese vaccine strain II (CVCC40202):

Carbosap and Canadian bison are $pXO2^{+}$ and share both the qPCR profile ($sap^{+}/cap^{+}/BclA^{+}$) and the 315 bp *BclA* amplicon with CVCC40202. Our assay cannot distinguish these strains from CVCC40202 using the current markers..

5. Relevance to China:

All wild-type strains of Chinese origin tested (Ames, Shikan-NIID, Ohio ACB, IAL 52, A0248, Vollum, HYU01, BA1035, 2000031021) produced a *BclA* amplicon of 558–675 bp, which is distinct from the 315 bp of CVCC40202 and the 729 bp of Sterne. The only wild-type strain giving a 315 bp amplicon was Canadian bison, which is not endemic to China. Thus, within the Chinese epidemiological context, the method reliably discriminates field isolates from vaccine strains.

Manuscript revisions: The in silico approach is now described in the Materials and Methods (lines 327–336), the results are presented in Results (lines 151–173), and the implications and limitations are discussed in Discussion (lines 187–212).

Minor Comments 1: Why is standard plasmid used for the sensitivity testing of Multiplex qPCR Assay instead of *B. anthracis* genomic DNA?

Response: Thank you for your thoughtful question. We used plasmid standards instead of B. anthracis genomic DNA for sensitivity testing for the following reasons:

1.Copy number uniformity across targets: In *B. anthracis*, the three target genes reside on different genetic elements: *sap* is on the chromosome (single copy per cell), cap is on the pXO2 plasmid (copy number may vary and is absent in acapsular strains), and *BclA* is on the chromosome (single copy). Therefore, genomic DNA cannot provide an equimolar ratio of the three targets, making it impossible to define a single "copy number" that applies equally to all targets. In contrast, our plasmid standards (pMD19-sap, pMD19-cap, pMD19-BclA) can be mixed at precisely known and equal copy numbers, allowing a fair and direct comparison of amplification efficiency and the determination of a unified limit of detection (LOD).

2. Avoidance of extraction-related variability: Genomic DNA preparations vary in purity and integrity, and fragmentation can disproportionately affect longer amplicons. Plasmids are small, circular, and can be prepared at very high purity and defined concentration, ensuring reproducibility across experiments.

3. Establishment of an absolute LOD: Reporting LOD in copies/μL (based on plasmid quantification) provides an absolute, instrument-independent performance metric that allows direct comparison with other qPCR assays. Using genomic DNA would only permit reporting in mass units (e.g., pg/μL), which is less comparable due to strain-to-strain variation in genome size and plasmid content.

Minor Comments 2: Line 252 and Line 187 in Fig 4， Fig 6, Chinese vaccine strain II CVCC40202 is not Sterne-based vaccine strain.Sterne-type vaccine strains belonged to

A.Br.001/002 subgroup while the CVCC40202 strain belonged to A.Br.008/009 subgroup.

Response: Thank you for your careful correction. We have reviewed the relevant literature and confirmed that, based on canSNP genotyping, the Chinese vaccine strain II (CVCC40202) belongs to the A.Br.008/009 subgroup, not the Sterne-type (A.Br.001/002)

lineage. This strain was originally introduced from the former Soviet Union and is classified as the Tsiankovskii-I strain, distinct from the Pasteur strain.

Accordingly, we have revised the manuscript to remove all references to "Sterne-based" for Chinese vaccine strain II. Specifically:

1. Figure 1 legend (line 450): The label "*B. anthracis Sterne*-based (Strain II) vaccine strain" has been replaced with "Chinese vaccine strain II".

2. Figure 4 legend (lines 467, 469-470): Both occurrences of "Sterne-based vaccine strain (Strain II)" have been replaced with "Chinese vaccine strain II ".

3.Figure 6 legend (line 487): The label "Sterne-based vaccine strain (Strain II)" has been replaced with "Chinese vaccine strain II ".

Response to Reviewer #3

We greatly appreciate your positive evaluation of our work and your constructive suggestions. We have carefully addressed each comment, and the manuscript has been substantially improved. Below is our point-by-point response. All changes are highlighted in the revised manuscript.

Major Comments

Comment 1： The validation section could be strengthened by an analysis of additional vaccine strains. Only two vaccine strains were included in the technical validation, and it would be beneficial to include data of how other vaccine strains might be identified by this two-step algorithmic approach. Additional analysis could be limited to in-silico analysis if strains are unavailable.

Response: Thank you for this constructive suggestion. We agree that testing additional vaccine strains would strengthen the validation. Because physical access to these strains is limited, we have performed a complementary in silico analysis using publicly available B. anthracis genome assemblies from the NCBI database.

We selected 19 complete genomes representing diverse canSNP subgroups, which include multiple vaccine strains from different lineages. Virtual PCR was performed using our primer/probe sets, and the BclA amplicon length was calculated directly from each genome.

How the two-step algorithm identifies other vaccine strains:

The multiplex qPCR profile (based on sap, cap, and BclA signals) allows immediate differentiation of vaccine strains that lack pXO2 (e.g., acapsular strains) from the pXO2^{-} positive Chinese vaccine strain II.

For vaccine strains that share the same qPCR profile (sap^{+}/cap^{+}/BclA^{+}) as CVCC40202, the *BclA* fragment length provides additional resolution. Among the tested genomes, all vaccine strains (except one rare genotype not found in China) produced a *BclA* amplicon size distinct from that of CVCC40202, ensuring reliable identification.

A minor limitation is that the Carbosap strain (pXO2^{+}, 315 bp) cannot be distinguished from Chinese vaccine strain II (CVCC40202) using these markers, but this genotype is not present in the Chinese epizootic landscape. Within the Chinese epidemiological context, the method reliably discriminates wild-type from vaccine strains.

The revised Method section is detailed in lines 327–336, the Results section in lines 151–173, and the Discussion section in lines 187–219.

Comment 2: The discussion could be enhanced by a robust review of previous work and where historical methods to differentiate have failed and how this assay in particular builds upon previous work or fulfills a regional gap in *B. anthracis* diagnostics. Please review literature on the topics covered in your manuscript published in the early 2000s. In particular, review manuscripts authored/coauthored by P Keim, et al, and L.B. Price, et al. highlight assays that differentiated between vaccine and virulent strains that were developed in the 1990s.

Response: Thank you for this important suggestion. We have substantially revised the Discussion to include a robust review of historical methods, specifically the pioneering work by Keim and colleagues (reference 12) from the late 1990s and early 2000s, as well as subsequent canSNP genotyping (15, 16).

1. We explicitly acknowledge that MLVA (12) could discriminate between vaccine and wild-type strains but required post-PCR fragment sizing using automated DNA sequencers, making it labor-intensive for routine diagnostics.

2. We also note that canSNP genotyping (15, 16) provides higher phylogenetic resolution but typically requires multiple separate reactions or specialized equipment, limiting its use in rapid screening.

3. To directly demonstrate the limitations of existing PCR-based assays, we performed simulated PCR using primer sets from WHO (1), Keim et al. (12), Ogawa et al. (13), and Zorigt et al. (14) on 19 publicly available B. anthracis genomes. These methods failed to differentiate vaccine strains carrying both pXO1 and pXO2 (e.g., the Chinese vaccine strain II, CVCC40202) from certain wild-type strains.

4. We then show that our two-step strategy (multiplex qPCR followed by conventional PCR and gel electrophoresis) overcomes this limitation for the vast majority of Chinese field isolates, while being simpler and more practical than earlier methods.

Manuscript revisions: These additions are now in the Discussion (lines 187–212).

Minor Comments

Minor comment 1: Page 2, Lines 43-45: Sentence is not complete and needs revision.

Response: We apologize for the incomplete sentence. It has been corrected in the revised manuscript (Introduction section, lines 45-46). The sentence now reads: "... the frequency of outbreaks varies, with most cases concentrated in rural rainfed systems, where it poses a major threat..."**.**

Minor comment 2: Page 2, line 47: Please clarify that pXO2, encoding the poly-D-glutamate, includes the cap gene of interest described here.

Response: Thank you for pointing this out. We have clarified in the revised manuscript (Introduction, line 48) that the pXO2 plasmid carries the cap gene cluster, which encodes the poly-D-glutamate capsule. The sentence now reads: "... pXO2, which carries the cap gene cluster (encoding the poly-D-glutamate capsule)." We believe this directly addresses your concern.

In this study, the primers and probes for the *cap* genes were designed within the *capA* region of the *B. anthracis* Ames strain. Furthermore, the primer and probe sequences used in this method have been successfully patented and are provided in Table 7 of the manuscript (Line 515).

Minor comment 3: Page 2, Lines 52-54: Recommend adding a citation to support statement of existing gap in diagnostics.

Response: Thank you for this suggestion. We have now performed simulated PCR experiments using the primer sets reported in several key studies (Keim et al., 2000, reference 12; Ogawa et al., 2015, reference 13; Zorigt et al., 2024, reference 14). The results clearly showed that these methods fail to differentiate vaccine strains carrying both pXO1 and pXO2 (e.g., CVCC40202) from certain wild-type strains. These citations are now included in the Discussion section (lines 187-212) to support our experimental comparison. The detailed results are presented there, and the diagnostic gap is therefore substantiated by our own data, not by the citations alone.

Minor comment 4: Page 3, lines 89-96: This section could be moved in its entirety to the methods section.

Response: Thank you for this thoughtful suggestion. We have carefully considered it. The paragraph in question presents the final optimized conditions (specific primer/probe concentrations and cycling parameters) that were derived from our optimization experiments. As such, we consider these to be results rather than part of the optimization process itself. The detailed optimization process, including the systematic variation of concentrations and temperatures, is already fully described in the Materials and Methods section. To maintain a clear logical flow presenting the final established assay conditions before the validation data, we would prefer to keep this paragraph in the Results section. To avoid any confusion, we have added a brief cross-reference to the Methods section at the beginning of the paragraph. We hope this addresses your concern and appreciate your understanding.

Minor comment 5: Figure 4, Table 1: The authors should comment on why the multiplex detecting *BclA* was negative for Sterne (34F2) and wild-type HC1, but the conventional PCR using the same primers for *BclA* resulted in a *BclA* amplicon.

Response: We have added a Note to the legend of Figure 4 (Line 473-476) and Table 1 (494-497) explaining this phenomenon:"For acapsular Sterne strain and HC1, the *BclA* gene was not detected by qPCR (Cq $\geq$ 35, scored as negative) due to nucleotide mismatches in the probe binding region (confirmed by sequencing), whereas the primer binding sites remain conserved, allowing conventional PCR to produce an amplicon."

Minor comment 6: Page 9, line 268: Is this assay intended to be used on cultured isolates or clinical materials (e.g., blood samples from infected individuals)? The authors could expand on the intended applications of this assay and how this diagnostic approach would not require culture enrichment.

Response: Thank you for your careful review. In addition to cultured isolates, this method is also applicable to the testing of clinical materials or environmental samples: First, perform a preliminary screening for *B. anthracis* in clinical materials or environmental samples using triple qPCR; if *B. anthracis* is detected, and the Cq value from the triple qPCR results is high

(Cq > 35), the sample must undergo culture enrichment and isolating, followed by *BclA* gene polymorphism analysis to distinguish between the wild-type and vaccine-type strains.

We sincerely thank both reviewers for their time and expertise. We believe that the revised manuscript has been substantially improved and hope it is now suitable for publication in *Microbiology Spectrum*.

Sincerely,

The authors

Re: Spectrum00192-26R1 (An Integrated Strategy of Multiplex qPCR and BclA Gene Length Polymorphism Analysis for Differentiating Bacillus anthracis Wild-Type and Vaccine Strains)

Dear Prof. Yonggang Qu:

Your manuscript has been accepted, and I am forwarding it to the ASM production staff for publication. Your paper will first be checked to make sure all elements meet the technical requirements. ASM staff will contact you if anything needs to be revised before copyediting and production can begin. Otherwise, you will be notified when your proofs are ready to be viewed.

Data Availability: ASM policy requires that data be available to the public upon online posting of the article, so please verify all links to sequence records, if present, and make sure that each number retrieves the full record of the data. If a new accession number is not linked or a link is broken, provide production staff with the correct URL for the record. If the accession numbers for new data are not publicly accessible before the expected online posting of the article, publication may be delayed; please contact ASM production staff immediately with the expected release date.

Publication Fees: For information on publication fees and which article types have charges, please visit our website. We have partnered with Copyright Clearance Center (CCC) to collect author charges. If fees apply to your paper, you will receive a message from no-reply@copyright.com with further instructions. For questions related to paying charges through RightsLink, please contact CCC at ASM_Support@copyright.com or toll free at +1-877-622-5543. CCC makes every attempt to respond to all emails within 24 hours.

ASM Membership: Corresponding authors may join or renew ASM membership to obtain discounts on publication fees. Need to upgrade your membership level? Please contact Customer Service at Service@asmusa.org.

Embargo Policy: A press release may be issued as soon as the manuscript is posted on the Spectrum Latest Articles webpage. The corresponding author will receive an email with the subject line "ASM Journals Author Services Notification" when the article is available online.

Cover Image Submissions: If you would like to submit a potential Cover Image, please email a file and a short legend to spectrum@asmusa.org. Please note that we can only consider images that (i) the authors created or own and (ii) have not been previously published. By submitting, you agree that the image can be used under the same terms as the published article. Image File requirements: TIF/EPS, 7.5 inches wide by 8.25 inches tall (at least 2,250 pixels wide by 2,475 pixels tall), minimum 300 dpi resolution (600 dpi preferred), RGB, and no figure elements, e.g., arrows or panel labels. The legend should be a short description of the image, 1-2 sentences recommended. Please download and use this interactive template in Adobe the https://journals.asm.org/pb-assets/html/asm-cover-sizing-tool.html to ensure that your proposed cover image meets our size requirements (https://journals.asm.org/pb-assets/pdf-text-excel-files/ASM-Interactive-Sizing-Cover-Template-1715689791.pdf).

The ASM Journals program strives for constant improvement in our submission and publication process. Please tell us how we can improve your experience by taking this quick Author Survey.

Thank you for submitting your paper to Spectrum.

Sincerely,
Cecilia Thompson
Editor
Microbiology Spectrum

Reviewer #2 (Comments for the Author):

The author has responded to the reviewers' comments one by one, and the manuscript has been significantly improved. I have no further comments.
