# Supplementary material for: An integrated strategy of multiplex qPCR and BclA gene length polymorphism analysis for differentiating Bacillus anthracis wild-type and vaccine strains
Source: Microbiol Spectr. 2026 Sep 1;14(10):e00192-26. doi: 10.1128/spectrum.00192-26 (PMC13688997; doi:10.1128/spectrum.00192-26)
Supplement: Supplementary file 2 [file spectrum.00192-26-s0001.docx]

**Supplementary Material**

**Supplementary Table S1. Strain designations and lane order for all simulated PCR gels.**

| **Lane** | **Strain** | **Description** |
| --- | --- | --- |
| MW | DNA marker | – |
| 1 | Ames | Wild‑type |
| 2 | Shikan‑NIID | Wild‑type |
| 3 | Ohio ACB | Wild‑type |
| 4 | IAL 52 | Wild‑type |
| 5 | A0248 | Wild‑type |
| 6 | Canadian bison | Wild‑type |
| 7 | Vollum | Wild‑type |
| 8 | HYU01 | Wild‑type |
| 9 | BA1035 | Wild‑type |
| 10 | 2000031021 | Wild‑type |
| 11 | A16R | Vaccine strain |
| 12 | Brazilian Vaccinal | Vaccine strain |
| 13 | acapsular Sterne strain 34F2 | Vaccine strain |
| 14 | V770‑NP1‑R | Vaccine strain |
| 15 | Tsiankovskii‑I | Vaccine‑related strain |
| 16 | Chinese vaccine strain II | Vaccine strain |
| 17 | STI1 | Vaccine strain |
| 18 | Pasteur | Vaccine strain |
| 19 | Carbosap | Vaccine strain |

**Supplementary Figure S1. Simulated PCR using the *BclA* primer pair designed in this study**

Using the forward primer AGTCCTGCTGGAAGTCCTAGTC and reverse primer ACCACCGATACCACCATTTACC, amplification of the *BclA* gene from 19 representative B. anthracis strains was performed. Wild-type strains (Ames, Shikan-NIID, Ohio ACB, IAL 52, A0248) and vaccine strains (A16R, Brazilian Vaccinal, acapsular Sterne strain 34F2, V770-NP1-R) produced amplicons of 558-729 bp. Wild-type strains (Vollum, HYU01, BA1035, 2000031021) and the Pasteur vaccine strain gave amplicons of 198-297 bp. The Tsiankovskii-I strain showed no amplification. Canadian bison (wild-type) and vaccine strains STI1 and Carbosap produced a 315 bp amplicon, identical to that of the Chinese vaccine strain II.

**Supplementary Figure S2. Simulated PCR using WHO recommended primers (*sap* gene)**

Using the forward primer CGCGTTTCTATGGCATCTCTTCT and reverse primer TTCTGCAGCTGGCGTTACAAAT, the *sap* gene (located on the chromosome) was amplified in all wild-type and vaccine strains, yielding a 639 bp product in all cases.

**Supplementary Figure S3. Simulated PCR using WHO recommended primers (pag gene)**

Using the forward primer TCCTAACACTAACGAAGTCG and reverse primer GAGGTAGAAGGATATACGGT, the *pag* gene (located on the pXO1 plasmid) was amplified in wild-type strains 1-9 and vaccine strains 11-16 and 19, but not in strains lacking pXO1.

**Supplementary Figure S4. Simulated PCR using WHO‑recommended primers (*cap* gene)**

Using the forward primer CTGAGCCATTAATCGATATG and reverse primer TCCCACTTACGTAATCTGAG, the *cap* gene (located on the pXO2 plasmid) was amplified in all wild-type strains and in vaccine strains 15, 16, 18, and 19.

Note: The three primer set recommended by WHO can distinguish wild-type strains from acapsular vaccine strains but cannot differentiate wild-type strains from the Chinese vaccine strain II (CVCC40202).

**Supplementary Figure S5. Simulated PCR using Keim primers (*VrrC*)**

Using the forward primer GAAGCAAGAAAGTGATGTAGTGGAC and reverse primer CATTTCCTCAAGTGCTACAGGTTC, the *VrrC* gene (chromosomal) was amplified in all wild-type and vaccine strains.

**Supplementary Figure S6. Simulated PCR using Keim primers (*pXO1-AAT*)**

Using the forward primer CAATTTATTAACGATCAGATTAAGTTCA and reverse primer TCTAGAATTAGTTGCTTCATATGGC, the *pXO1-AAT* marker (located on pXO1) was amplified in wild-type strains 1-9 and vaccine strains 11-16 and 19.

**Supplementary Figure S7. Simulated PCR using Keim primers (*pXO2-AT*)**

Using the forward primer TCATCCTCTTTTAAGTCTTGGGT and reverse primer GTGTGATGAACTCCGACGACC, the *pXO2-AT* marker (located on pXO2) was amplified in all wild-type strains and in vaccine strains 15, 16, 18, and 19.

Note: The three primer set described by Keim et al. (2000) can distinguish wild-type strains from acapsular vaccine strains but cannot differentiate wild-type strains from the Chinese vaccine strain II.

**Supplementary Figure S8. Simulated PCR using Ogawa primers (*BA_5031*)**

Using the forward primer CGATGTAAATTCGGCACTGGATCTTC and reverse primer TTCTATCATTCTTAGTGGAATGTGG, the *BA_5031* gene (chromosomal) was amplified in all wild-type and vaccine strains.

**Supplementary Figure S9. Simulated PCR using Ogawa primers (*pag*)**

Using the forward primer CCTTGTGGCAGCTTATCCGA and reverse primer GTAGATTGGAGCCGTCCCAG, the *pag* gene (pXO1) was amplified in wild-type strains 1-9 and vaccine strains 11-16 and 19.

**Supplementary Figure S10. Simulated PCR using Ogawa primers (*cap*)**

Using the forward primer TCATCCGGATCCAGGAGCAATGAG and reverse primer GCAGGTAAAATACCTGTTCTTTCTG, the *cap* gene (pXO2) was amplified in all wild-type strains and in vaccine strains 15, 16, 18, and 19.

Note: The three primer set of Ogawa et al. (2015) can distinguish wild-type strains from acapsular vaccine strains but cannot differentiate wild-type strains from the Chinese vaccine strain II.

**Supplementary Figure S11. Simulated PCR using Zorigt primers (BA5361)**

Using the forward primer CCCCAATATATTAACAATAGAC and reverse primer GTTTTTGAATGTTTTTTGGTTC, the *BA5361* gene (chromosomal) was amplified in all wild-type and vaccine strains.

**Supplementary Figure S12. Simulated PCR using Zorigt primers (*pag*)**

Using the forward primer CCAGACCGTGACAATGATGG and reverse primer TGGTAACACGTTGTAGATTGGAG, the *pag* gene (pXO1) was amplified in wild-type strains 1-9 and vaccine strains 11-16 and 19.

**Supplementary Figure S13. Simulated PCR using Zorigt primers (*capA*)**

Using the forward primer CACGTAAATGATATTGCACAAGTGC and reverse primer GGCAGATATTATTGTGGGACACC, the *capA* gene (pXO2) was amplified in all wild-type strains and in vaccine strains 15, 16, 18, and 19.

Note: The three primer set of Zorigt et al. (2024) can distinguish wild-type strains from acapsular vaccine strains but cannot differentiate wild-type strains from the Chinese vaccine strain II.
